# A Poly-γ-Glutamic Acid/ε-Polylysine Hydrogel: Synthesis, Characterization, and Its Role in Accelerated Wound Healing

**DOI:** 10.3390/gels11040226

**Published:** 2025-03-22

**Authors:** Jiaqi Li, Yuanli Huang, Yalu Wang, Qianqian Han

**Affiliations:** 1 National Institutes for Food and Drug Control, Beijing 100050, China; cpuljq1996@126.com (J.L.); yusnlihuang@nifdc.org.cn (Y.H.); w18263546952@163.com (Y.W.); 2 Department of Pharmaceutics, China Pharmaceutical University, Nanjing 211198, China; 3 School of Medical Devices, Shenyang Pharmaceutical University, Benxi 117004, China

**Keywords:** poly-γ-glutamic acid, ε-polylysine, hydrogel, wound healing

## Abstract

Wound healing is a complex biological process involving inflammation, proliferation, and remodeling phases. Effective healing is essential for maintaining skin integrity, driving the need for advanced materials like hydrogels, known for their high water retention and tunable mechanical properties. In this study, we synthesized a biocompatible composite hydrogel composed of γ-polyglutamic acid (γ-PGA) and ε-polylysine (ε-PL) through a Schiff base reaction, forming a stable crosslinked network. Its physicochemical properties, including rheological behavior and swelling capacity, were systematically evaluated. Biocompatibility was assessed via in vitro hemolysis and cytotoxicity assays, and in vivo testing was performed using a full-thickness skin defect model in Sprague Dawley (SD) rats to evaluate wound-healing efficacy. The PGA-PL hydrogel demonstrated excellent physicochemical properties, with a maximum swelling ratio of 65.6%, and biocompatibility as evidenced by low hemolysis rates (<5%) and high cell viability (>80%). It promoted wound healing by inhibiting the inflammatory response, reducing levels of the inflammatory cytokine IL-6, enhancing angiogenesis, and accelerating collagen deposition. The hydrogel showed complete biodegradation within 21 days in vivo without inducing a significant inflammatory response and significantly accelerated wound healing, achieving an 86% healing rate within 7 days compared to 67% in the control group. The PGA-PL composite hydrogel exhibits excellent mechanical strength and biocompatibility, and its effective wound-healing capabilities lay the groundwork for future development and optimization in various tissue engineering applications.

## 1. Introduction

Wound healing is a complex biological process involving three overlapping but distinct phases: inflammation, proliferation, and remodeling [1,2,3]. During the inflammatory phase, immune cells such as neutrophils and macrophages are recruited to the wound site to clear debris and pathogens, initiating the healing process [4,5]. The proliferative phase is characterized by fibroblast activation, angiogenesis, and the extracellular matrix (ECM) deposition, while the remodeling phase involves the ECM maturation and the scar tissue formation [3]. Effective wound healing is crucial for maintaining skin barrier function and preventing complications, and advancing the development of sophisticated wound-healing materials is a key focus in biomedical research [5,6,7].

Three-dimensional polymeric networks termed hydrogels possess the ability to retain substantial quantities of water and show potential in wound-healing applications. With extensive water retention, biocompatibility, and adjustable mechanical properties, these materials serve as suitable substitutes for natural ECM in supporting tissue regeneration [8,9]. Traditional wound dressings like gauze and cotton often show poor hemostasis effects and limited antibacterial properties and can cause reinjury during dressing changes. In contrast, hydrogels have been widely applied in wound dressings, drug delivery systems, and tissue engineering scaffolds, demonstrating superior performance in maintaining wound moisture, absorbing exudates, and promoting tissue repair [8,10,11]. Recent hydrogel research has focused on enhancing antibacterial properties, mechanical strength, and bioactivity. For instance, Zhao et al. developed an antibacterial, antioxidant, and electroactive injectable hydrogel with self-healing properties, which significantly accelerated wound healing [12]. Similarly, hydrogels incorporating bioactive molecules such as growth factors and cytokine-free strategies have demonstrated improved angiogenesis and tissue regeneration [8,13].

Currently, various hydrogels with similar polypeptide-based compositions have been explored for wound-healing applications. Traditional chitosan-based hydrogels exhibit effective antibacterial properties, yet require acidic conditions for dissolution [14]. Hyaluronic acid-based hydrogels demonstrate favorable biocompatibility while remaining susceptible to rapid enzymatic degradation in vivo [15]. Alginate hydrogels, though readily formed by ionic crosslinking, may stimulate pro-inflammatory cytokine secretion and prolong the inflammatory phase [8]. Recent studies on polypeptide-based hydrogels often involve toxic crosslinkers or complex processing conditions that compromise biocompatibility and practical applications [16,17,18,19].

γ-polyglutamic acid (γ-PGA), a naturally occurring anionic biopolymer produced by the Bacillus species, has garnered substantial attention in both materials science and biomedicine [20,21]. Initially discovered in the capsule of Bacillus anthracis, γ-PGA can be produced through microbial fermentation with different stereostructures (γ-D-PGA, γ-L-PGA, and γ-D/L-PGA) [20]. Its remarkable properties, including excellent biocompatibility, biodegradability, and water retention capacity, make it particularly suitable for biomedical applications [22]. The molecular configuration of γ-PGA determines its physicochemical behavior. The hydrogen bonding between molecules contributes to water retention capacity, which underlies its application in wound-dressing studies [22,23]. Additionally, γ-PGA has demonstrated promising results in promoting cell adhesion, proliferation, and tissue regeneration [24]. For instance, studies showed that γ-PGA-based scaffolds could effectively enhance wound healing by maintaining appropriate moisture levels and facilitating tissue repair [25].

ε-Polylysine (ε-PL), an FDA-approved natural cationic polymer consisting of L-lysine units connected through amide linkages between ε-amino and α-carboxyl groups, has emerged as a promising biomaterial for tissue engineering applications [26]. Its excellent biocompatibility, biodegradability, and unique molecular structure make it particularly suitable for biomedical applications. Recent studies have demonstrated that ε-PL-based materials possess favorable properties for tissue regeneration, including good cell adhesion promotion and ECM production enhancement [13,26,27]. In the context of wound healing, ε-PL has shown remarkable potential in maintaining an appropriate wound microenvironment and promoting tissue repair [11,13,27]. When incorporated into hydrogel systems, ε-PL improves the mechanical properties and biological performance of the composite materials through its abundant amino groups and unique molecular structure.

In this study, we developed a novel biocompatible hydrogel by combining γ-PGA and ε-PL through a Schiff base reaction, which formed a stable crosslinked network structure. The physicochemical properties of the hydrogel were systematically investigated, including rheological behavior and swelling properties. The mechanical properties of the hydrogel could be readily adjusted by varying the ratio of γ-PGA to ε-PL. The biocompatibility of the composite hydrogel was evaluated through in vitro cell experiments and in vivo subcutaneous implantation. Furthermore, we explored its wound-healing efficacy using a full-thickness skin defect model in rats, analyzing the wound closure rate, histological changes, and the tissue regeneration process. The composite hydrogel demonstrated excellent biocompatibility and wound healing promotion, suggesting its potential as an ideal biomaterial for tissue repair. From a practical perspective, the γ-PGA/ε-PL hydrogel system offers advantages in scalable production. Both γ-PGA and ε-PL are produced through fermentation processes at low cost, enabling industrial-scale manufacturing. EDC and NHS are widely used industrial crosslinkers that provide favorable biocompatibility and cost efficiency. Their combination with the Schiff base crosslinking mechanism simplifies manufacturing without the need for complex equipment or harsh conditions. Overall, the raw materials and processing requirements are relatively low compared to those of existing tissue repair materials. This study provided valuable insights into the development of novel biodegradable hydrogels for tissue engineering and regenerative medicine.

## 2. Results and Discussion

### 2.1. Synthesis of Hydrogels

**Figure 1:**
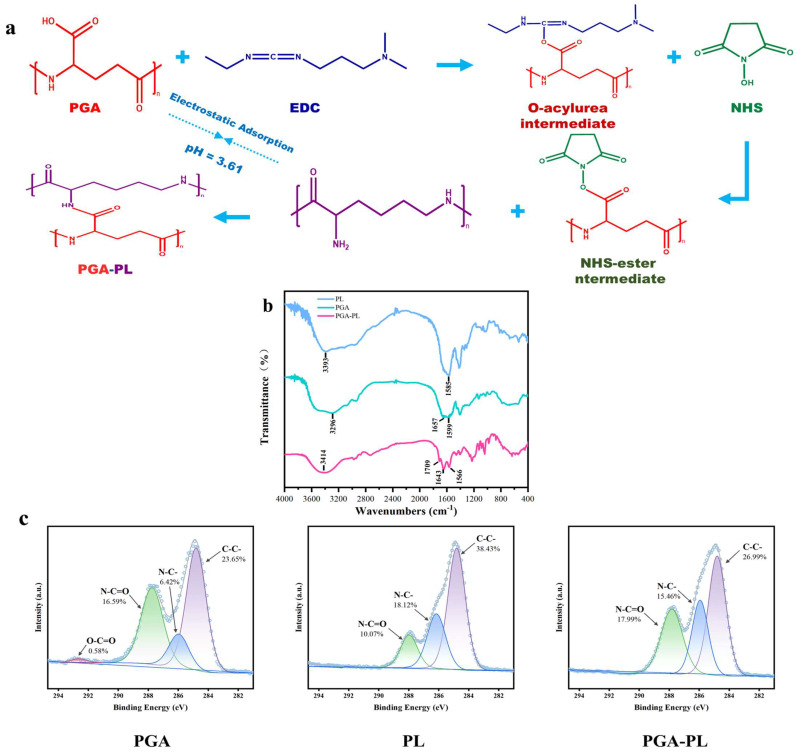
(**a**) Preparation principle of PGA-PL hydrogel; (**b**) FT-IR spectra of PGA, PL, and PGA-PL hydrogels; (**c**) High-resolution C1s spectra of XPS detection for PGA, PL, and PGA-PL hydrogels.

The synthesis of PGA-PL hydrogels was achieved through a synergistic combination of electrostatic adsorption and chemical crosslinking, the latter mediated by an EDC/NHS coupling system (Figure 1a). Initially, electrostatic interactions between the negatively charged carboxyl groups (-COO^−^) of γ-PGA and the positively charged amino groups (-NH_3_^+^) of ε-PL led to the formation of a preliminary physically crosslinked network, establishing a foundation for subsequent hydrogel development. To enhance the structural stability of the hydrogel, 1-ethyl-3-(3-dimethylaminopropyl) carbodiimide (EDC) and N-hydroxysuccinimide (NHS) were introduced into the system to facilitate chemical crosslinking. During this process, EDC first activated the carboxyl groups (-COOH) of PGA molecules to form O-acylisourea intermediates, which subsequently reacted with NHS to generate NHS ester intermediates. These NHS ester intermediates then underwent rapid nucleophilic substitution with the primary amino groups (-NH_2_) of PL, resulting in the formation of stable amide bonds (-CONH-).

Considering that the O-acylisourea intermediates generated during EDC activation are highly unstable and prone to hydrolysis, which could compromise crosslinking efficiency, NHS was pre-incubated at −20 °C to ensure its uniform distribution and optimal interaction with PGA. This low-temperature pre-treatment step facilitated the rapid formation of more uniformly distributed, stable, and highly reactive NHS ester intermediates upon the subsequent addition of EDC, thereby ensuring efficient coupling with PL amino groups. This optimized process resulted in the formation of a robust chemically crosslinked network between PGA and PL molecules, significantly enhancing the structural homogeneity and mechanical properties of the hydrogel network.

**Figure 2:**
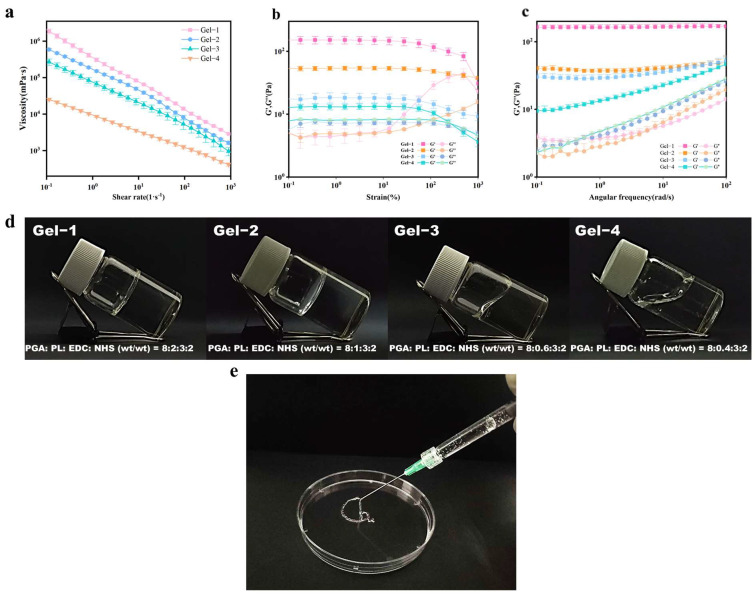
(**a**) The viscosity test of PGA-PL hydrogels with different proportions; (**b**) curves of storage modulus (G′) and loss modulus (G″) of PGA-PL hydrogels with strain; (**c**) curves of storage modulus (G′) and loss modulus (G″) of PGA-PL hydrogels with angular frequency; (**d**) images of hydrogel samples; (**e**) injectability of PGA-PL hydrogels.

This synthesis strategy, harnessing the synergistic effects of both physical and chemical crosslinking mechanisms, produced stable PGA-PL hydrogels with enhanced structural uniformity, mechanical strength, and operational stability. Notably, as illustrated in Figure 2d, the gelation state and flowability of the PGA-PL hydrogels were significantly influenced by the varying ratios of PGA to PL. Specifically, Gel-1 and Gel-2, which contained the highest proportions of PL, exhibited a jelly-like consistency with minimal flowability, while Gel-3 and Gel-4 demonstrated increased fluidity as the PL content decreased, suggesting potential injectable applications—a characteristic that was later confirmed through rheological analysis (Figure 2a–c). The robust properties of these hydrogels, combined with their tunable mechanical characteristics, provided a strong foundation for their potential applications in various biomedical fields.

### 2.2. Physicochemical Characterization of Hydrogels

#### 2.2.1. Chemical Structure Analysis of Hydrogels

The absorption spectra of PGA, PL, and Gel-2 hydrogels were characterized by Fourier-Transform Infrared Spectroscopy (FTIR) (Figure 1b). The shift in the IR spectrum of the PGA-PL hydrogel at 3414 cm^−1^, compared to γ-PGA and ε-PL, is attributed to the O-H bond stretching vibration, indicating an enhancement of interactions between the polymer chains. The newly generated absorption peak at 1709 cm^−1^ corresponds to the C=O stretching vibration, demonstrating the formation of a carbonyl structure and the presence of amide bonding. This formation resulted from the reaction between the amino group in polyglutamic acid and the carboxyl group in polylysine. The peaks at 1643 cm^−1^ and 1566 cm^−1^ are attributed to the N-H bond bending vibration and the -CONH- bond vibration, respectively, suggesting that the crosslinking process occured during the formation of the -CONH- bond. This further confirms the enhancement of inter-amino group interactions and supports the validity of the crosslinking reaction. In conclusion, the results of FTIR Spectroscopy confirmed that the PGA-PL hydrogels are crosslinked through the reaction of the amino group in polyglutamic acid with the carboxyl group in polylysine, resulting in the formation of an amide bond.

X-Ray Photoelectron Spectroscopy (XPS) analysis provided additional confirmation of the hydrogel crosslinking mechanism, as shown in Figure 1c. In γ-PGA, the O-C=O component (carboxyl groups) decreased from 0.58% of the C1s spectral area to undetectable levels in the hydrogel, indicating near-complete conversion to amide bonds. Concurrently, the N-C=O component (amide bonds) increased from 16.59% to 17.99%, while the C-N component decreased from 18.12% in PL to 15.46%, confirming amino group consumption. Complementary XPS and FTIR analyses confirmed the successful formation of chemically crosslinked PGA-PL hydrogels through the Schiff base reaction between PGA carboxyl and PL amino groups.

#### 2.2.2. Rheological Analysis of Hydrogels

Rheological characteristics were investigated to assess the practical applications of hydrogels. As illustrated in Figure 2a, all samples exhibited non-Newtonian fluid behavior, characterized by a decrease in shear viscosity with an increasing shear rate, which is indicative of shear-thinning properties. These properties provided a solid foundation for the injectability of the hydrogels, thereby confirming the feasibility of their in vivo injection applications. Figure 2b presents the stress scanning spectra of the PGA-PL hydrogels. The energy storage modulus (G′) and loss modulus (G″) exhibited minimal variation within the strain range of 0.1% to 10%, suggesting that the hydrogel demonstrated resilience to large strains and maintained a robust three-dimensional network structure. Figure 2c shows the frequency scanning spectra of the PGA-PL hydrogels, revealing that G′ consistently exceeds G″ within the frequency range of 1–100 rad/s. This observation was corroborated by the stress scan results. These results confirmed that the hydrogel possessed a strong three-dimensional structure and viscoelastic properties. The increase in shear viscosity and G′ with rising PL concentration was attributed to enhanced crosslinking with PGA. This led to increased intermolecular entanglement and stronger polymer interactions. This enhancement contributed to the stability of the three-dimensional network structure of the hydrogels. Consequently, among the hydrogel samples, Gel-1 exhibited optimal mechanical properties, while Gel-4 demonstrated exemplary flow characteristics. These findings provided significant theoretical and practical support for the application of hydrogels in various fields.

Rheological analysis revealed the mechanical characteristics of the PGA-PL hydrogel system. The structural stability of the hydrogel network enhanced through a dual-crosslinking mechanism involving electrostatic adsorption and EDC/NHS coupling. Existing hydrogel systems exhibited notable mechanical constraints. CMC/ε-PL hydrogels showed significant stress relaxation in CP43 and CP45 variants, indicating structural instability during application [7]. PPM hydrogels [23] and fibrin-based matrices [28] revealed mechanical instability over extended periods. Dual-crosslinking strategies enhanced hydrogel stability, per recent studies on HA/ε-PL hydrogels utilizing combined enzymatic and chemical crosslinking [13]. The PGA-PL hydrogels maintained stable viscoelastic properties across dynamic loading via physical and chemical crosslinking networks. Enhanced structural integrity emerged from appropriate crosslinking density and balanced hydrophilic–hydrophobic interactions between PGA and PL components, enabling potential applications in wound-dressing materials where sustained mechanical stability is crucial.

#### 2.2.3. Swelling Ratio of Hydrogels

**Figure 3:**
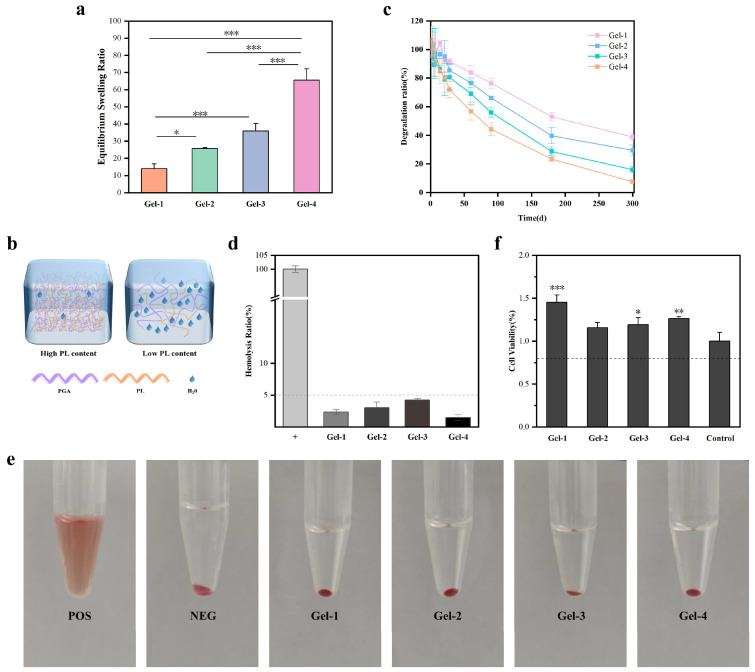
(**a**) Swelling ratio of the hydrogel (* *p* < 0.05, and *** *p* < 0.001, data without asterisks indicate no significant difference); (**b**) schematic diagram of hydrogel network structures and swelling behaviors under different PGA/PL ratios; (**c**) in vitro degradation of the hydrogel; (**d**) hemolysis experiments of the hydrogel; (**e**) images of hemolysis performance of the POS (the positive control), NEG (the negative control), and Gel-1, Gel-2, Gel-3, and Gel-4 groups; (**f**) cytotoxicity of the hydrogel (* *p* < 0.05, ** *p* < 0.01, and *** *p* < 0.001 compared to the control group; data without asterisks indicate no significant difference).

The swelling behavior of hydrogels is critically important for the effective absorption of wound exudate and the subsequent healing process. It serves as a key factor in promoting wound recovery. The swelling test (Figure 3a) showed that the hydrogel exhibited exceptional swelling properties, attributed to the high hydrophilicity of both polyglutamic acid and polylysine, enhancing the hydration characteristics of the hydrogel. Moreover, it was observed that the swelling properties of the hydrogel significantly decreased with an increase in the ratio of polylysine. This phenomenon is explained by the increased crosslinking degree with a higher polylysine ratio, resulting in a more compact network structure. This compactness restricts the penetration of water molecules into the hydrogel’s interior, thereby diminishing the swelling rate. Additionally, while polylysine possesses a certain degree of hydrophilicity, an increase in the content of ε-polylysine strengthens the hydrogen bonds between the -NH_2_ groups and the water molecules within the polymer chain. This leads to the formation of a more robust hydrogen bond network, which further limits the free entry of water molecules and consequently reduces the swelling capacity of the hydrogel. Specifically, the highest ratio of polylysine in Gel-1 resulted in a significant degree of crosslinking, forming a relatively dense three-dimensional network structure, which yielded a swelling rate of only 14.0%. Conversely, as the ratio of polylysine decreases, the degree of crosslinking in Gel-2, Gel-3, and Gel-4 also diminishes, resulting in a more relaxed network structure. These hydrogels demonstrated an enhanced ability to change volume in the hydrated state, facilitating interactions with water molecules, particularly in Gel-4. The expansion of the number and diameter of hydration channels within the three-dimensional network effectively increased the permeability of water molecules, thereby significantly improving the swelling performance of the network, with the swelling rate reaching 65.6%. This observation was further supported by porosity analysis.

#### 2.2.4. In Vitro Degradation of Hydrogels

In the in vitro degradation experiments (Figure 3c), PGA-PL hydrogels with varying crosslinking densities were evaluated in PBS solution at 37 °C for 300 days. Gel-1, with the highest crosslinking density, retained 38.8% of its initial mass after 300 days, while Gel-4, with the lowest crosslinking density, retained only 7.52%. The tightly packed network of Gel-1 restricted water molecule penetration, significantly slowing the hydrolysis rate. Its low swelling ratio and high mechanical strength also maintained structural integrity and physical properties during storage. For example, Gel-1 maintained 92.78% of its initial mass at 21 days, whereas dopamine-modified eugenol hydrogels reported by Zhang et al. exhibited approximately 60% mass retention at 22 days [29]. The improved stability was further demonstrated in extended studies, with Gel-1 retaining 38.8% of its initial mass through 300 days, in contrast to PGA-based hydrogels with polyhydrazide crosslinks reported by Liu et al. [30], which underwent complete degradation within the same timeframe under similar conditions. These degradation properties, along with the robust mechanical properties, make it suitable for long-term biomedical applications such as drug delivery carriers or tissue engineering scaffolds. Gel-4, with its low crosslinking density, formed a loose network and exhibited a high swelling ratio, increasing its susceptibility to water molecule infiltration. This led to lower mechanical strength and a higher propensity for degradation and structural collapse, limiting its use in scenarios requiring long-term stability. However, its rapid degradation behavior and high hydration capacity proved effective in short-term applications, such as acute wound dressings or short-term drug delivery systems, where it could quickly absorb exudates or release drugs to meet clinical needs.

The tunable degradation behavior of PGA-PL hydrogels highlights their potential for applications in drug delivery and tissue engineering. Modulating the crosslinking density allows precise control over the degradation rate and drug release kinetics, meeting diverse therapeutic requirements. Low crosslinking density hydrogels like Gel-4, with rapid degradation properties, are well suited for short-term drug release systems, such as immediate drug delivery in acute infections or post-operative pain management. High crosslinking density hydrogels like Gel-1, with slow degradation characteristics, are ideal for long-term drug release systems, such as sustained drug delivery in chronic diseases like diabetes or cancer. The high mechanical strength and adjustable degradation behavior of PGA-PL hydrogels also make them excellent candidates for tissue engineering scaffolds, supporting cell growth and tissue regeneration.

### 2.3. In Vitro Biocompatibility of Hydrogels

It is imperative that materials utilized in the biomedical field exhibit optimal biocompatibility. To comprehensively evaluate the hemocompatibility of PGA-PL hydrogels, a hemolysis test was conducted using hydrogel samples extracted at 0.2 g/mL in normal saline and tested against pure water (positive control) and normal saline (negative control), employing fresh rabbit blood anticoagulated with 0.38% trisodium citrate solution and diluted in saline at a 4:5 ratio. The induction of blood hemolysis was determined to assess the hemocompatibility of PGA-PL hydrogels. The hemolysis test results (Figure 3d,e) showed that all hydrogel groups had ratios below 5%, which was within the acceptable range for biomaterials [22]. In the cell viability evaluation (Figure 3f), Human Dermal Fibroblast (HDF) reached activity values above 80% when cultured with PGA-PL hydrogels. The experimental results confirmed these materials were biologically compatible. The excellent biocompatibility of the hydrogel can be attributed to its constituent materials and synthesis process. Both γ-PGA and ε-PL are natural polymers with well-documented biocompatibility and non-immunogenicity [18,19,20]. Additionally, the crosslinking agents EDC and NHS are widely recognized for their mild reaction conditions and favorable biosafety profiles in biomaterial preparation [23]. Moreover, the utilization of water as the sole solvent during synthesis further ensures the environmental friendliness and biocompatibility of the final hydrogel product. These intrinsic properties and the green synthesis approach synergistically contributed to the formation of a biocompatible hydrogel system suitable for biomedical applications.

### 2.4. In Vivo Biocompatibility of Hydrogels

**Figure 4:**
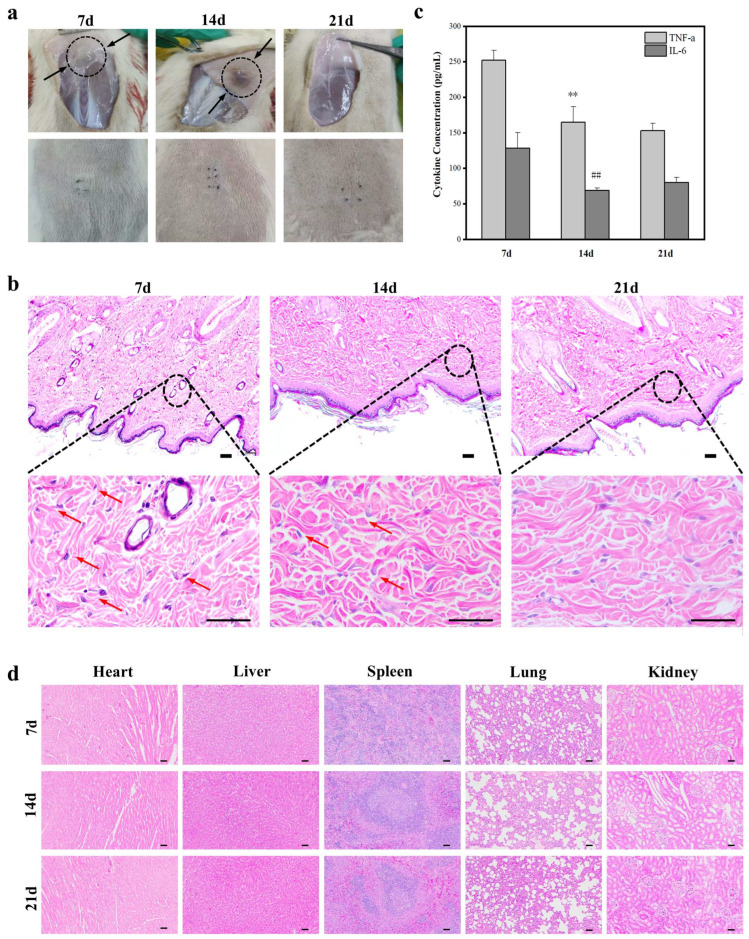
In vivo degradability. (**a**) Degradation of dorsal implantation in rats; (**b**) sagittal rat skin HE staining (Scale bar: 50 μm). The dotted circle indicates the location of the magnified area, with arrows denoting infiltrating lymphocytes; (**c**) TNF-α content ** *p* < 0.01 compared to day 7; data without asterisks indicate no significant difference) and IL-6 content ## *p* < 0.01 compared to day 7; data without asterisks indicate no significant difference); (**d**) corona pathological sections of major organs (Scale bar: 50 μm).

The biodegradability of PGA-PL hydrogels is essential for their application in wound healing, as it ensures the gradual replacement of the material with regenerated tissue. To assess the biocompatibility and biodegradability of hydrogels in vivo, we implanted the hydrogels subcutaneously in rats. Throughout the 21-day study, all rats remained healthy and were euthanized at 7, 14, and 21 days post-implantation. The major organs (heart, liver, spleen, lungs, and kidneys) of the rats, along with the dorsal skin at the implantation site, were collected for further analysis. The implanted regions (Figure 4a) remained free from tissue damage, inflammatory response, and purulent secretion at days 7, 14, and 21 post-surgery. The surgical sites remained normal in appearance, and the surrounding fur grew normally. The hydrogel material underwent progressive volume reduction throughout the observation period and achieved complete breakdown by day 21, demonstrating its in vivo degradation rate. The in vivo degradation rate was consistent with previously reported injectable hydrogels. Wang et al. reported complete degradation of carboxymethyl chitosan/oxidized alginate hydrogels by day 28 [7], while Liu et al. demonstrated that mussel-inspired dual-crosslinking hyaluronic acid hydrogels completely degraded between days 14 and 21 [13].

Subsequently, we performed hematoxylin–eosin (H&E) staining to assess the host’s inflammatory response to the hydrogel. As shown in Figure 4b, at day 7 post-implantation, the tissues surrounding the implantation site exhibited a mild inflammatory response, primarily characterized by sparse lymphocytic infiltration, which represented a normal immune response to the implanted material. The inflammation was notably lower compared with previously reported similar hydrogel systems. For instance, Yang et al. observed pronounced inflammatory cell infiltration with significant neutrophil accumulation in their injectable self-healing hyaluronic acid hydrogels during the initial week post-implantation [10]. No neutrophil infiltration or tissue necrosis was observed, indicating the absence of an acute inflammatory response. The surrounding tissues maintained their normal architecture, with intact appendages, including hair follicles and sebaceous glands, showing no signs of congestion or necrosis, demonstrating excellent biocompatibility of the hydrogel system. By day 14 post-implantation, the inflammatory response had markedly decreased, as evidenced by significantly reduced lymphocytic infiltration and improved tissue organization. At day 21 post-implantation, the inflammatory response was minimal, with tissue morphology comparable to that of normal tissue and well-defined epidermis and dermis layers. These observations indicated that the hydrogel induced only a mild initial inflammatory response and was well tolerated by the host, demonstrating excellent in vivo biocompatibility. We also measured the levels of Tumor Necrosis Factor-alpha (TNF-α) and Interleukin-6 (IL-6) in rat skin, and the results corroborated these observations (Figure 4c). Furthermore, we evaluated the systemic toxicity of the hydrogel on days 7, 14, and 21, respectively, and found no toxicity in the major organs of the rats (Figure 4d). These findings suggested that the subcutaneously implanted hydrogel exhibited good in vivo biocompatibility and biodegradability.

### 2.5. In Vivo Wound-Healing Activity

**Figure 5:**
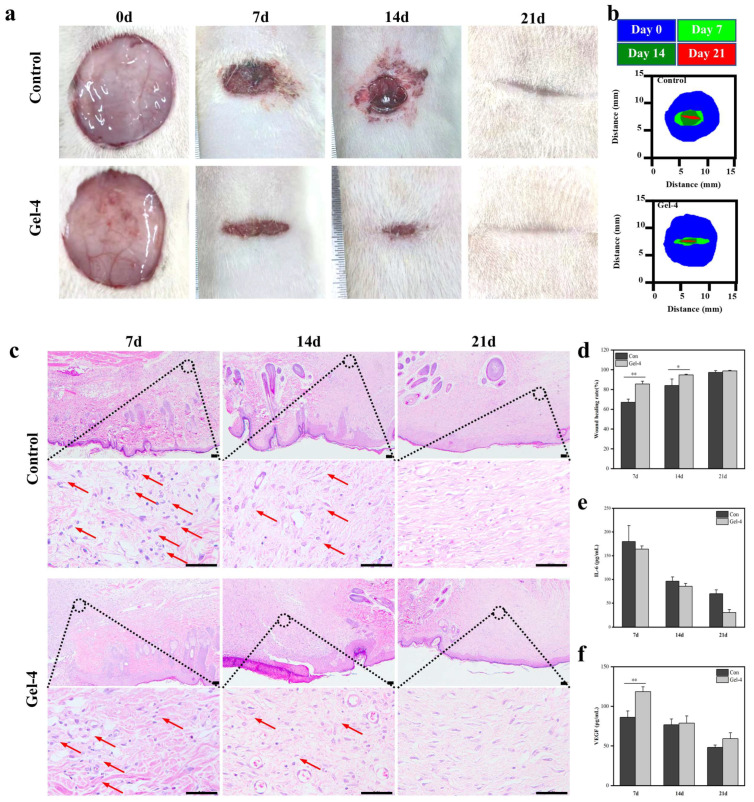
(**a**) Representative images of wound beds at each time point during wound healing; (**b**) changes in the wound area over time; (**c**) sagittal representative HE staining of wound tissues in each group on day 7, day 14, and day 21 (Scale bar: 50 μm). The arrows denote infiltrating lymphocytes.; (**d**) wound-healing rate at each time point; (**e**) IL-6 content of wound beds at each time point during wound healing; (**f**) VEGF content of wound beds at each time point during wound healing. (* *p* < 0.05, ** *p* < 0.01; data without asterisks indicate no significant difference).

To demonstrate the effect of this hydrogel based on γ-polyglutamate and ε-polylysine in promoting wound healing, we comprehensively evaluated the wound-healing potential of the γ-PGA/ε-PL hydrogel by establishing a wound model. The results showed that the hydrogel could significantly accelerate the wound-healing process (Figure 5a,b). At day 7, the healing rate of the hydrogel group reached about 86%, while that of the control group was only 67% (Figure 5d). Histological analysis showed that the hydrogel-treated group exhibited a more ordered tissue structure and less inflammatory cell infiltration (Figure 5c), which was also supported by the determination of IL-6 content, indicating that it effectively inhibited the inflammatory response (Figure 5e).

**Figure 6:**
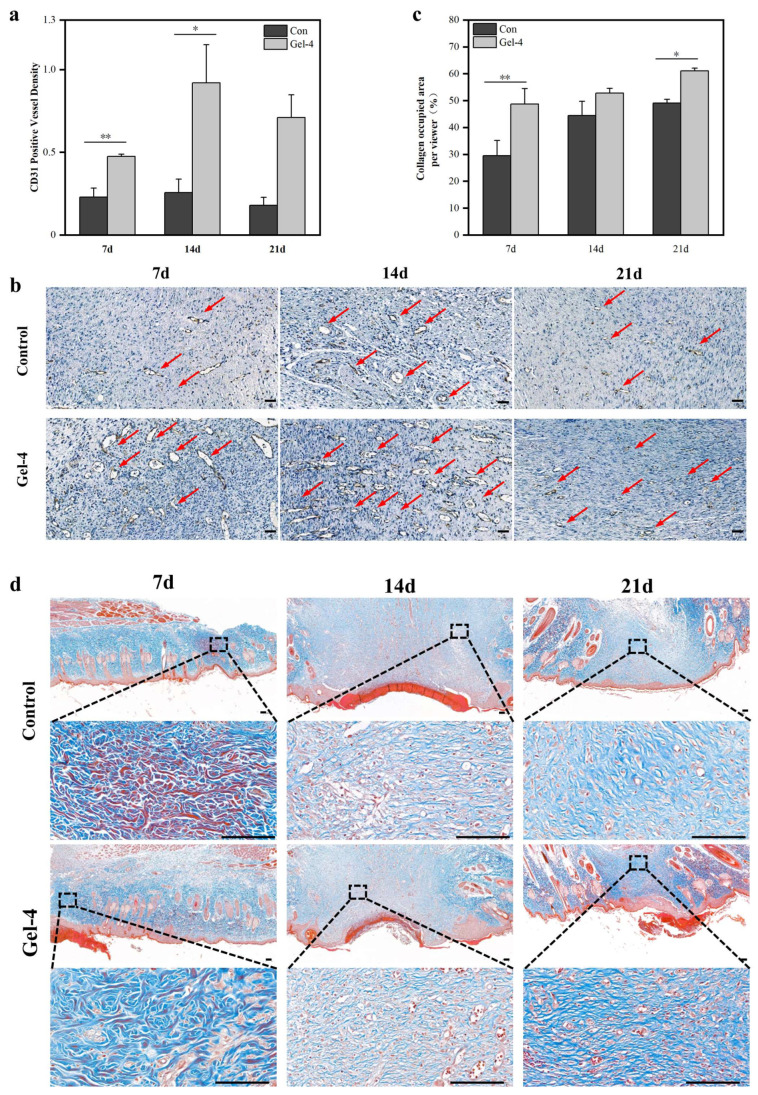
(**a**) CD31 content of wound beds at each time point during wound healing. (* *p* < 0.05; ** *p* < 0.01); (**b**) sagittal representative CD 31 immunohistochemical images of wound tissues on day 7, 14, and 21 (Scale bar: 50 μm). The arrows denote newly formed blood vessels; (**c**) CD 31 immunohistochemical quantitative analysis of the vessel for wound tissues on day 7, 14, and 21; (**d**) sagittal representative Masson trichrome staining images of wound tissues on day 7, 14, and 21 (Scale bar: 100 μm). (* *p* < 0.05, ** *p* < 0.01; data without asterisks indicate no significant difference).

The Vascular Endothelial Growth Factor (VEGF) is a key factor in the early stage of wound healing and can promote the generation of new blood vessels. The expression level of the Cluster of Differentiation 31 (CD31) marks the maturation and functionalization of blood vessels. Compared with the control group, the hydrogel group showed significant differences in promoting the expression of VEGF (Figure 5f) and CD31 (Figure 6a). On day 7, the VEGF level in the hydrogel group was significantly increased, indicating that the hydrogel promoted the expression of early angiogenic factors, accelerated the formation of new blood vessels, and provided the necessary oxygen and nutrition for wound healing. Previous studies suggest that the enhanced angiogenesis might be related to the potential activation of PI3K/Akt and HIF-1α/VEGF signaling pathways, where PGA’s degradation products could upregulate VEGF expression [31,32,33,34]. By day 14, with the initial wound healing, CD31 expression in the hydrogel group significantly increased, while VEGF levels gradually decreased. This indicated that in the later stage of healing, the number of new blood vessels decreased and the blood vessels gradually matured. The enhanced function and stability of the blood vessels is clearly shown by the immunohistochemical map (Figure 6b).

Masson trichrome staining showed that the collagen content of the hydrogel group was significantly higher than that of the control group on days 7 and 21, indicating that the PGA-PL hydrogel could effectively promote collagen deposition (Figure 6c,d). Collagen was the main structural protein in wound healing, supporting tissue reconstruction, and the increased collagen deposition indicated accelerated tissue regeneration. The observed collagen deposition might be attributed to the hydrogel’s unique physicochemical properties, where the positively charged PL facilitated ordered collagen fiber arrangement through electrostatic interactions, while the hydrogel’s three-dimensional structure supported collagen crosslinking and reorganization [35]. Previous studies have demonstrated that PGA degradation products activate the transforming growth factor-β (TGF-β)/Smad signaling pathway, upregulating key collagen synthesis enzymes such as proline hydroxylase and collagen synthase [30,31,32]. These degradation products also suppress matrix metalloproteinase (MMP) activity, reducing collagen degradation and further enhancing collagen deposition [30]. Existing studies suggest that PGA treatment can significantly increase collagen content and promote its maturation, which is essential for tissue reconstruction and scar prevention [31,35]. Additionally, the hydrogel’s ability to maintain moderate collagen deposition could help prevent excessive scar formation while supporting tissue integrity [1].

Therefore, the PGA-PL hydrogels promoted angiogenesis by stimulating early VEGF expression, which ensured nutrient supply for cell proliferation and tissue repair. Following initial wound healing, the increased CD31 expression accelerated vascular maturation, providing adequate blood supply throughout the healing process. Moreover, accelerated collagen deposition enhanced the stability and integrity of the regenerated tissue. Notably, the hydrogel’s anti-inflammatory activity might synergistically enhance angiogenesis. The PGA-PL hydrogel was shown to modulate the nuclear factor–kappa B (NF-κB) signaling pathway and Toll-like receptor 2/Toll-like receptor 6/cluster of differentiation 14 (TLR2/TLR6/CD14) immune response axis, promoting macrophage polarization toward an anti-inflammatory phenotype [35,36,37]. These M2-polarized macrophages actively secreted pro-angiogenic factors, such as VEGF and TGF-β, which enhanced vascularization and supported tissue regeneration [29,34]. Early VEGF expression was stimulated by the hydrogel, facilitating blood vessel formation and providing essential nutritional support for cell proliferation. Furthermore, increased CD31 expression promoted blood vessel maturation, ensuring sufficient blood supply throughout the healing process. Through its dual role in modulating inflammation and promoting angiogenesis, the hydrogel established a favorable microenvironment for tissue regeneration. In conclusion, the PGA-PL hydrogel accelerated wound healing by inhibiting inflammation, promoting angiogenesis, and accelerating collagen deposition, providing a promising therapeutic strategy for clinical applications in tissue engineering and regenerative medicine.

## 3. Conclusions

A composite hydrogel of PGA and PL was developed through a combination of electrostatic interactions and chemical crosslinking. The material exhibited excellent physicochemical properties in rheological and swelling tests, demonstrating favorable biodegradability and biocompatibility, as confirmed by in vitro hemolysis and cytotoxicity assays. In an SD rat full-thickness skin wound model, the hydrogel significantly reduced levels of the inflammatory cytokine IL-6, effectively suppressing the inflammatory response. Moreover, it accelerated wound healing by promoting angiogenesis and collagen deposition. These findings highlight the unique multifunctional potential of the PGA-PL hydrogel as an innovative tissue repair material.

This study revealed a significant correlation between the PGA-PL ratio and the hydrogel’s rheological and mechanical properties. A decrease in PL concentration enhanced fluidity and induced observable changes in rheological behavior, as confirmed by rheological tests. Adjusting the PGA-PL ratio can optimize the hydrogel’s mechanical properties, providing a solid foundation for designing PGA-PL hydrogels tailored to specific biomedical needs. Unlike traditional hydrogels such as hyaluronic acid, whose mechanical properties are primarily determined by molecular weight and are difficult to precisely control, PGA-PL hydrogels offer the advantage of tunable mechanical properties through component ratio adjustment. This enables their use in diverse scenarios. For example, increasing the proportion of PL enhances the hydrogel’s mechanical strength, making it suitable for applications requiring sustained mechanical support, such as load-bearing tissue engineering or 3D printing biomaterials. Conversely, reducing the PL content improves the hydrogel’s fluidity and injectability, which is beneficial for applications like acute wound dressings or short-term drug delivery systems.

Despite these promising properties, several limitations remain. Highly crosslinked hydrogels exhibit excellent stability during long-term storage, but their mechanical strength and degradation rates may limit their use in certain dynamic environments, such as load-bearing tissue engineering or joint injection materials. Additionally, although the hydrogel demonstrated good biocompatibility both in vitro and in vivo, its performance in more complex or prolonged implantation scenarios, such as dynamic mechanical environments or chronic wound-healing applications, remains to be fully explored. Further research is needed to assess its long-term stability and functionality under these conditions. Future studies should focus on addressing these challenges to advance the broader application of PGA-PL hydrogels in biomedical fields.

## 4. Materials and Methods

### 4.1. Materials

γ-polyglutamic acid (γ-PGA, MW > 700 KDa) was purchased from Lushang Freda Pharmaceutical Co., Ltd (Jinan, China). 1-(3-dimethylaminopropyl)-3-ethylcarbodiimide hydrochloride (EDC, MW: 191.7) was purchased from Shanghai Titan Scientific Co., Ltd. (Shanghai, China). N-hydroxysuccinimide (NHS, MW: 115.09) and ε-poly-L-lysine (ε-pl, MV 2000–5000) were purchased from Shanghai Aladdin Biochemical Technology Co., Ltd. (Shanghai, China). Phosphate-buffered saline (PBS) was obtained from Pricella (Wuhan, China). The TNF-α Elisa kit, IL-6 Elisa kit and VEFG Elisa kit were obtained from Solarbio (Beijing, China). All chemicals were used as received from suppliers. Sprague Dawley (SD) rats were obtained from the Animal Resource Center of the National Institute for Food and Drug Control, with experimental animal use permission No. SYXK (Beijing, China) 2022-0014.

### 4.2. Synthesis of Hydrogels

PGA-PL hydrogels were synthesized via EDC/NHS-mediated polymerization as follows: γ-polyglutamic acid (γ-PGA) was first dissolved in an MES (2-(N-morpholino) ethanesulfonic acid) buffer solution (pH = 3.61) at room temperature (25 °C) under stirring until a homogeneous solution was formed, referred to as Solution A. Similarly, ε-polylysine (ε-PL) was dissolved in the same MES buffer under identical conditions to prepare Solution B. Subsequently, Solution B was added dropwise into the stirring Solution A until a uniform mixture was obtained, which was designated as Solution C. To optimize the activation of the carboxyl groups on γ-PGA, N-hydroxysuccinimide (NHS) was added to Solution C, and the mixture was incubated at −20 °C for 30 min to ensure sufficient activation of the intermediate NHS esters, enabling subsequent coupling with the amino groups of ε-PL. Finally, 1-ethyl-3-(3-dimethylaminopropyl)carbodiimide (EDC) was added to Solution C with rapid stirring to produce the PGA-PL hydrogel. The resulting hydrogels were then subjected to structural and functional characterization to evaluate their performance for biomedical applications.

### 4.3. Fourier-Transform Infrared Spectroscopy (FTIR) Characterization

The spectral characterization of the samples was conducted using FTIR analysis. Prior to measurement, the purified PGA-PL hydrogel (thoroughly washed to remove unreacted agents) was freeze-dried to a constant weight. The resulting materials were thoroughly ground and subsequently blended with KBr to prepare thin pellets. FTIR spectra were recorded on a Thermo Scientific Nicolet iS 10 spectrometer across the wavenumber range of 400–4000 cm^−1^.

### 4.4. X-Ray Photoelectron Spectroscopy (XPS) Test

XPS analysis was performed using a Thermo Scientific ESCALAB 250Xi instrument (Waltham, MA, USA) with Al Kα radiation (1486.6 eV). The γ-PGA and ε-PL, as well as the freeze-dried PGA-PL hydrogel (thoroughly washed to remove unreacted agents), were analyzed after mounting on conductive carbon tape. Survey spectra were collected over a binding energy range of 0–1350 eV. Data processing and elemental analysis were conducted using Thermo Scientific Avantage 6.6.0 software.

### 4.5. Rheological Analysis

Rheological measurements were performed on an HR10 rheometer (New Castle, DE, USA) at 37 °C. Dynamic oscillatory tests were conducted to determine the storage modulus (G′) and loss modulus (G″). The samples were subjected to frequency sweeps from 0.1 to 100 rad/s while maintaining 1% strain amplitude. Additionally, steady-state flow measurements were carried out by varying the shear rate from 0.1 to 1000 S^−1^ to assess the viscosity behavior.

### 4.6. Swelling Ratio Test

The swelling ratios of hydrogels were measured by weighing the freeze-dried hydrogels and recording the weight as W_0_. Next, the freeze-dried hydrogels were soaked in PBS (pH 7.2) at room temperature. To measure the swelling behavior, we removed the hydrogel samples from the PBS solution at predetermined intervals. The surface moisture was carefully eliminated using filter paper, and these samples were subsequently weighed (W_1_). The swelling ratio (%) of hydrogel was calculated using the following equation:

Swelling ratio (%) = (W_1_ − W_0_)/W_0_ × 100%

### 4.7. In Vitro Degradation of Hydrogels

The freeze-dried hydrogel samples were weighed and recorded as W_0_. The samples were then immersed in PBS (pH 7.2) at 37 °C with continuous shaking. At predetermined intervals, the hydrogel samples were retrieved from the PBS solution, rinsed with deionized water, and freeze-dried. The samples were then weighed (recorded as W_1_). The weight remaining (%) of the hydrogel was calculated using the following equation:

Weight Remaining (%) = W_1_/W_0_ × 100%

### 4.8. Hemolysis Ratios

The hemolysis test used diluted rabbit blood prepared with anticoagulant citrate dextrose and normal saline. The hydrogel samples were extracted in normal saline and tested with pure water (positive control) and normal saline (negative control). All samples were incubated at 37 °C and centrifuged. The optical density of the supernatant was measured at 545 nm for hemolysis rate calculation.

Hemolysis ratio (%) = (OD_sample_ − OD_negative_)/(OD_positive_ − OD_negative_) × 100%

### 4.9. In Vitro Biocompatibility of Hydrogels

Human Dermal Fibroblast (HDF) cells were obtained from Guangdong Boxi Biotechnology Co., Ltd., Zhongshan, China. The cells were maintained in Dulbecco’s Modified Eagle Medium (DMEM, Gibco, Agawam, MA, USA) containing 10% Fetal Bovine Serum (FBS) and 1% antibiotics. To obtain the extract, hydrogels were immersed in culture medium at 37 °C for 24 h. The cells were then seeded into 96-well plates (5 × 10^4^ cells/mL, 0.1 mL per well) with the prepared extracts, using fresh medium as a control. Cultures were maintained in a 5% CO_2_ atmosphere at 37 °C. After a 24 h culture period, Cell Counting Kit-8 (CCK-8) reagent was added to the wells and incubated for 2 h.

### 4.10. In Vivo Degradation of Hydrogels

Animal experiments were performed in accordance with protocols approved by the Local Ethical Committee and Laboratory Animal Administration Rules of China and the National Institute for Food and Drug Control Medical Research Ethics Committee. All the SD rats were purchased from the Animal Resource Center of the National Institute for Food and Drug Control, with experimental animal use permission No. SCXK (Beijing) 2022-0002. All the materials used for animal experiments were sterilized by ultraviolet radiation.

To assess the tissue compatibility and in vivo degradation capacity of the hydrogel, Gel-1 was selected for implantation due to its higher crosslinking density and material content, which would represent the maximum possible tissue response. Although hydrogels with lower PL content demonstrated injectable properties, the highly crosslinked Gel-1 required surgical implantation due to its limited flowability. Nine male SD rats (150–200 g) underwent pentobarbital anesthesia (i.p.) prior to the experiment. The back hair was shaved, and a 2 mm incision was created for hydrogel implantation. The rats were euthanized at days 7, 14, and 21 post-operation. Tissues from the implant site and major organs were excised for H&E staining. The stained sections were examined using a Leica DM IL LED microscope. Skin tissue samples were analyzed for TNF-α and IL-6 levels by ELISA.

### 4.11. In Vivo Wound Healing

Male SD rats (150–200 g) underwent pentobarbital anesthesia (i.p.). After back hair removal with an electric razor, a full-thickness skin defect (20 mm) was created on the dorsal region using a biopsy punch. The rats (*n* = 5 per group) were assigned to either hydrogel treatment or blank control groups. To assess the in vivo wound-healing activity of the hydrogel, Gel-4 was chosen for its superior flow characteristics, which ensured consistent wound surface coverage and tissue contact, thereby reducing the risk of experimental bias caused by incomplete wound contact. The experimental group received hydrogel placement on the wound surface, while the control group remained untreated. Gauze bandages were applied to all wounds. After surgery, the rats were housed individually. Wound dimensions were measured and photographed at 0, 7, 14, and 21 days. Tissue specimens collected at days 7, 14, and 21 were fixed in 4% paraformaldehyde for histological examination. The fixed sections were processed for H&E staining to evaluate morphological changes and IHC staining to detect wound-healing markers. Collagen deposition was analyzed by Masson’s trichrome staining, and IL-6 levels were measured to assess the inflammatory response during wound healing.

### 4.12. Statistical Analysis

All experiments were independently repeated at least three times (n ≥ 3) and presented as mean ± standard deviation. Statistical significance between the results was determined by one-way ANOVA and Student’s *t*-test. *p* < 0.05 was considered to be statistically significant (* *p* < 0.05, ** *p* < 0.01, and *** *p* < 0.001).

## Data Availability

The data presented in this study are available in this article.
